# Parallel algorithms for large-scale biological sequence alignment on Xeon-Phi based clusters

**DOI:** 10.1186/s12859-016-1128-0

**Published:** 2016-07-19

**Authors:** Haidong Lan, Yuandong Chan, Kai Xu, Bertil Schmidt, Shaoliang Peng, Weiguo Liu

**Affiliations:** 1 School of Computer Science and Technology, Shandong University, Shunhua Road 1500, Jinan, Shandong, China; 2 Johannes Gutenberg University, Mainz, Germany; 3 School of Computer Science, National University of Defense Technology, Changsha, Hunan, China

**Keywords:** Smith-Waterman, Dynamic programming, Pairwise sequence alignment, Multiple sequence alignment, Xeon Phi clusters

## Abstract

**Background:**

Computing alignments between two or more sequences are common operations frequently performed in computational molecular biology. The continuing growth of biological sequence databases establishes the need for their efficient parallel implementation on modern accelerators.

**Results:**

This paper presents new approaches to high performance biological sequence database scanning with the Smith-Waterman algorithm and the first stage of progressive multiple sequence alignment based on the ClustalW heuristic on a Xeon Phi-based compute cluster. Our approach uses a three-level parallelization scheme to take full advantage of the compute power available on this type of architecture; i.e. cluster-level data parallelism, thread-level coarse-grained parallelism, and vector-level fine-grained parallelism. Furthermore, we re-organize the sequence datasets and use Xeon Phi shuffle operations to improve I/O efficiency.

**Conclusions:**

Evaluations show that our method achieves a peak overall performance up to 220 GCUPS for scanning real protein sequence databanks on a single node consisting of two Intel E5-2620 CPUs and two Intel Xeon Phi 7110P cards. It also exhibits good scalability in terms of sequence length and size, and number of compute nodes for both database scanning and multiple sequence alignment. Furthermore, the achieved performance is highly competitive in comparison to optimized Xeon Phi and GPU implementations. Our implementation is available at https://github.com/turbo0628/LSDBS-mpi.

## Background

Calculating similarity scores between a given query protein sequence and all sequences of a database and computing multiple sequence alignments are two common tasks in bioinformatics. Both tasks include iterative calculations of pairwise local alignments as a basic building block. This can lead to high runtimes for large-scale input data sets. Since biological sequence databases are continuously growing, finding fast solutions is of high importance. An approach to reduce associated runtimes is the implementation of basic alignment algorithms on parallel computer architectures [1–3]. More recently, the usage of modern massively parallel accelerator architectures such as CUDA-enabled GPUs has gained momentum [4]. In this paper we are investigating how a Xeon Phi-based compute cluster can be used as a computational platform to accelerate alignment algorithms based on dynamic programming for two applications:

(i) databases scanning of protein sequence databases with the Smith-Waterman algorithm, and
(ii) distance matrix computation for multiple sequence alignment (i.e. the first stage of the popular ClustalW heuristic).

Three levels of parallelization are required in order to exploit the compute power available in a cluster of Xeon Phis. Parallelization within a Xeon Phi is usually based on the “scale-and-vectorize” approach: scaling across the up to 61 cores requires the usage of several hundred threads while exploiting the 512-bit wide vector units requires SIMD vectorization within each core. Recent examples of efficient parallelization on Xeon Phis include scientific computing [5], bioinformatics [6–10], and database operations [11]. Furthermore, parallelization between Xeon Phis adds another level of message passing based parallelism. This level needs to consider data partitioning, load balancing, and task scheduling. The accelerator-based approach is motivated by the fact that the performance of many-core architectures is growing. For example, the 2nd generation Xeon Phi processor named “Knight’s Landing” has already been announced.

The rest of this paper is organized as follows. The “Related work” Section provides important background information about the Xeon Phi programming model, pairwise and multiple sequence alignment, and hardware accelerated alignment algorithms. Our single-node parallel algorithms are presented in the “Algorithms on a single node” Section. The “Cluster level data parallelization” Section describes our cluster-level parallelization. Section “Results and discussion” evaluates performance. Some conclusions are drawn in Section “Conclusion”.

## Related work

### Programming models on Xeon Phi coprocessor

Xeon Phi is a coprocessor connected via the PCI express (PCIe) bus to a host CPU. From a hardware perspective, it contains up to 61 86 compatible cores. Each core features a 512-bit vector processing unit (VPU) based on a new instruction set. The cache hierarchy contains a L1 data cache of size 32 KB and a 512 KB per core L2 cache. The cores are connected via a bidirectional ring bus which enables L2 cache coherence based on a directory based protocol. Each core can execute up to four threads at the same time.

Assuming a Xeon Phi with 61 usable cores running at 1.238 GHz, we can determine the peak performance for 32-bit integer (integer arithmetic is commonly used for sequence alignment calculations) operations as follows: 16 (#SIMD lanes) × 1 integer operation × 1.238 GHz × 61 (#cores) = 1.208 Tera integer operations per second.

From a software perspective, three programming models can be used in order to harness the compute power of the Xeon Phi: (i) native model, (ii) offload model, and (iii) symmetric model. In this paper, we have chosen the offload model. In this model, code sections and data can be offloaded from the host CPU to the Xeon Phi. Using OpenMP pragmas, offload regions can be specified. When encountering such a region during program execution, the necessary data transfers between host and Xeon Phi are performed and the code inside the (parallelized) region is executed on the Xeon Phi.

### Pairwise sequence alignment and database search

The database search application considered in this paper scans a protein sequence database using a single protein sequence as a query (similar to BLASTP). Different to the BLASTP heuristic, we calculate the score of an optimal local alignment between the query and each subject sequence using the Smith-Waterman algorithm with affine gap penalties (instead of a seed-and-extend approach). The subject sequences are ranked in terms of this score. Actual alignments are only computed for the top-ranked database sequences which only takes a negligible amount of time in comparison to the score-only search procedure. Note that the score-only Smith-Waterman computation can be performed in linear space and quadratic time with respect to the length of the alignment targets.

Consider two protein sequences *Q* and *S* and length *q* and *s*, respectively. We want to compute the score of an optimal local alignment of *Q* and *S* with respect to a given scoring scheme consisting of a gap opening penalty *α*, a gap extension penalty *β* and an amino acid substitution matrix *sbt*(). The well-known Smith-Waterman algorithm solves this problem by computing a dynamic programming matrix iteratively based on the following recurrence relations:

1 $$\begin{array}{@{}rcl@{}} H_{A}(i,j)&=&max\{0,E(i,j),F(i,j),H_{A}(i-1,j-1)\\ &&+sbt(Q[i],S[j])\}\\ E(i,j) &=& max\{H_{A}(i,j-1)-\alpha,E(i,j-1)-\beta\}\\ F(i,j) &=& max\{H_{A}(i-1,j)-\alpha,F(i-1,j)-\beta\} \end{array} $$

The iterative computation of theses matrices is started with the initial values: *H*_*A*_(*i*,0)=*H*_*A*_(0,*j*)=*E*(*i*,0)=*F*(0,*j*)=0 for all 0<=*i*<=*q*, 0<=*j*<=*s*.

### Progressive multiple sequence alignment

The time complexity of computing an optimal multiple alignment of more than two sequences grows exponentially in terms of the number of input sequences. Thus, heuristic approaches with polynomial complexities must be used in practice for large inputs to approximate the (generally unknown) optimal multiple alignment.

**Fig. 1:**
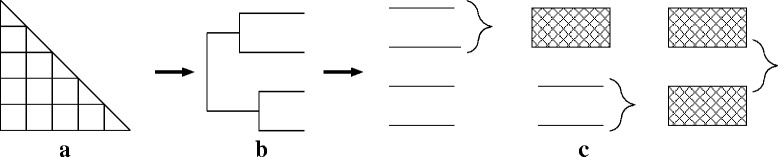
Illustration of the three stages of progressive multiple alignment (see text for details)
- *Guide tree*: Using the distance matrix computed in the previous step is taken as an input to compute an evolutionary tree using the neighbor-joining method [14].
- *Progressive alignment*: Following the branching order of the tree a multiple sequence alignment is build progressively.

The multiple (protein) sequence alignment application considered in this paper is the first stage of the popular ClustalW heuristic [12]. ClustalW is based on the classical progressive alignment approach [13] featuring a 3-step pipeline (see Fig. 1):

- *Distance matrix*: For each input sequence pair, a distance values is computed based on the Smith-Waterman algorithm

### Hardware accelerated alignment algorithms

We briefly review some previous work on accelerating pairwise alignment (based on Smith Waterman) and progressive multiple sequence alignment (based on ClustalW) on a number of parallel computer architectures. A number of SIMD implementations have been designed in order to harness the vector units of common multi-core CPUs (e.g. [15–21]) or the the Cell/BE (e.g. [22, 23]). Recent years has seen increased interests in acceleration of sequence alignment on massively parallel GPUs. Initially, programming these graphics chips for bioinformatics application still required programming with shaders using languages such as OpenGL [24]. The release of CUDA in 2007 made the usage GPUs for general purpose computing more accessible and subsequently a number of CUDA-enabled Smith-Waterman implementation have been presented in recent years [4, 25–33]. A number of MPI-based solutions for progressive multiple sequence alignments are targeted towards PC clusters [34–37]. Another attractive architecture for sequence analysis are FPGAs [38–41] which are based on reconfigurable hardware. However, in comparison to the other mentioned architectures, FPGAs are often less accessible and generally more difficult to program.

The solution in this paper is based on a cluster of Xeon Phis. Compared to common CPUs, a Xeon Phi contains significantly more cores and often a wider vector unit. Different from CUDA-enabled GPUs, a Xeon Phi provides x86 compatibility, which often simplifies the implementation process. Nevertheless, achieving near-optimal performance is still a challenge which needs to be addressed by parallel algorithm design and efficient implementation. In this paper we demonstrate how this can be done for protein sequence database search and distance matrix computation for multiple sequence alignment.

Compared to our previously presented LSBDS [9], we introduce the following new contributions in this paper:

- We have designed new algorithms which can handle searching tasks for large-scale protein databases on Xeon Phi clusters.
- We have designed new algorithms for calculating large-scale multiple sequence alignments on Xeon Phi clusters.
- We have implemented our multiple sequence alignment algorithm using the offload model to make full use of the compute power of both the multi-core CPUs and the many-core Xeon Phi hardware.

## Methods

### Algorithms on a single node

#### Protein sequence database search

We have observed two facts: (1) protein sequence database search has inherent data parallelism; (2) each VPU on Xeon Phi can execute multiple integer operations in an SIMD parallel way efficiently. Based on these two facts, we have partitioned the database search process on a single node into two data parallel parts: device level and thread level. The device level data parallel part is encoded on the host CPU. It splits the subject database into multiple batches that can be distributed to CPU and Xeon Phi devices. The thread level data parallel part is used to process data batches locally. In order to support search tasks for large-scale databases, we have designed a dynamic data distribution framework to distribute these batches to both the host CPU device and the Xeon Phi devices. In order to solve the performance loss problem for searching long query sequences, we have also proposed a multi-pass algorithm where long query sequences are partitioned into multiple short subsequences for consecutive searching passes. We have presented more implementation details of our algorithm in [9].

#### MSA

The distance matrix computation stage of ClustalW is typically a major runtime bottleneck. Thus, in our work we have only concentrated on designing a parallel algorithm for this stage. ClustalW bases the distance computation between two protein sequences on the following concept [24]:

##### **Definition1**.

Consider two sequences *S*_*i*_,*S*_*j*_∈*S*={*S*_1_,…*S*_*n*_}. The following equation defines their distance *d*(*S*_*i*_,*S*_*j*_):

$$\begin{array}{@{}rcl@{}} d(S_{i}, S_{j}) &=& 1 - \frac{nid(S_{i}, S_{j})}{min\{l_{i}, l_{j}\}} \end{array} $$

whereby *nid*(*S*_*i*_,*S*_*j*_) is defined as the number of exact matches in an optimal local alignment between *S*_*i*_ and *S*_*j*_. *l*_*i*_ (*l*_*j*_) is the length of *S*_*i*_ (*S*_*j*_).

**Fig. 2:**
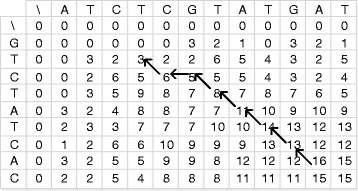
An example of how to compute the *nid*-value in the traceback procedure. The matrix *H*_*A*_(*i,j*) is shown for a linear gap penalty *α*=1, and a substitution score +3 for the exact match and −1 otherwise. The *nid*-value here is five

The value *nid*(*S*_*i*_,*S*_*j*_) can be calculated in the Smith-Waterman traceback procedure by counting the number of exact character matches. Figure 2 illustrates this method. However, this direct method does not work well for long sequences and large-scale datasets because it needs to store the whole DP matrix. In order to solve this problem, we have adapted the method presented in [24] to do the *nid*-value computation on the Xeon Phi architecture. That is we have used the following definition and theorem to calculate the *nid*-value without doing the actual traceback.

##### **Definition2**.

Consider two protein sequences *S*_1_ and *S*_2_, affine gap penalties *α*, *β*, and substitution matrix *sbt*. The matrix *N*_*A*_(*i,j*) (1≤*i*≤*l*_1_,1≤*j*≤*l*_2_) is defined in terms of the following recurrence relations:

$$\begin{array}{@{}rcl@{}} N_{A}(i,j) &=& \left\{ \begin{array}{l} 0,~~~~~~~~~~~~~~~~~~~\text{if}~H_{A}(i,j)=0\\\\ N_{A}(i-1,j-1)+m(i,j),\\~~~~~~~~~~~~~~~~~~~~~~~\text{if}~H_{A}(i,j)=H_{A}(i-1,j-1)\\ ~~~~~~~~~~~~~~~~~~~~~~~~~~~~~~~~~~~~~~~~~~~+sbt(S_{1}[i], S_{2}[j])\\\\ N_{E}(i,j),~~~~~~~~~\text{if}~H_{A}(i,j)=E(i,j)\\\\ N_{F}(i,j);~~~~~~~~~\text{if}~H_{A}(i,j)=F(i,j) \end{array} \right. \end{array} $$

where

$$\begin{array}{@{}rcl@{}} m(i,j) &=& \left\{ \begin{array}{l} 1,~~~~~~~~~~~~~~~~~\text{if}~S_{1}[i]=S_{2}[j]\\ 0;~~~~~~~~~~~~~~~otherwise \end{array} \right.\\ N_{E}(i,j) &=& \left\{ \begin{array}{l} 0,~~~~~~~~~~~~~~~~~\text{if}~ j=1\\ N_{A}(i,j-1),~~\text{if}~E(i,j)=H_{A}(i,j-1)-\alpha\\ N_{E}(i,j-1);~~\text{if}~E(i,j)=E(i,j-1)-\beta \end{array} \right.\\ N_{F}(i,j) &=& \left\{ \begin{array}{l} 0,~~~~~~~~~~~~~~~~~\text{if}~i=1\\ N_{A}(i-1,j),~~\text{if}~F(i,j)=H_{A}(i-1,j)-\alpha\\ N_{F}(i-1,j);~~\text{if}~F(i,j)=F(i-1,j)-\beta \end{array} \right. \end{array} $$

It can be shown that

$$nid(S_{1},S_{2})=N_{A}(i_{max},j_{max}) $$

where (*i*_*max*_,*j*_*max*_) denote the coordinates of the maximum value in the corresponding pairwise local alignment DP matrix *H*_*A*_.

**Fig. 3:**
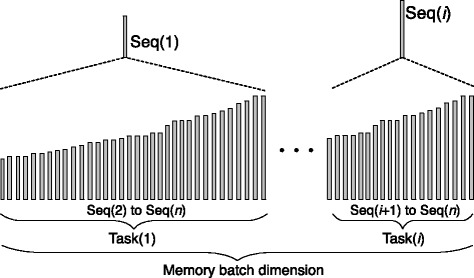
Illustration of our task partitioning scheme

**Fig. 4:**
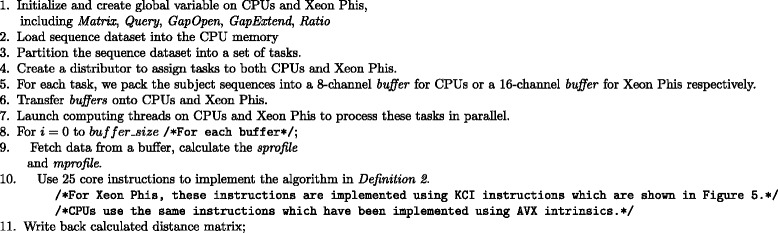
The pseudo-code of our MSA algorithm framework on a single computing node

**Fig. 5:**
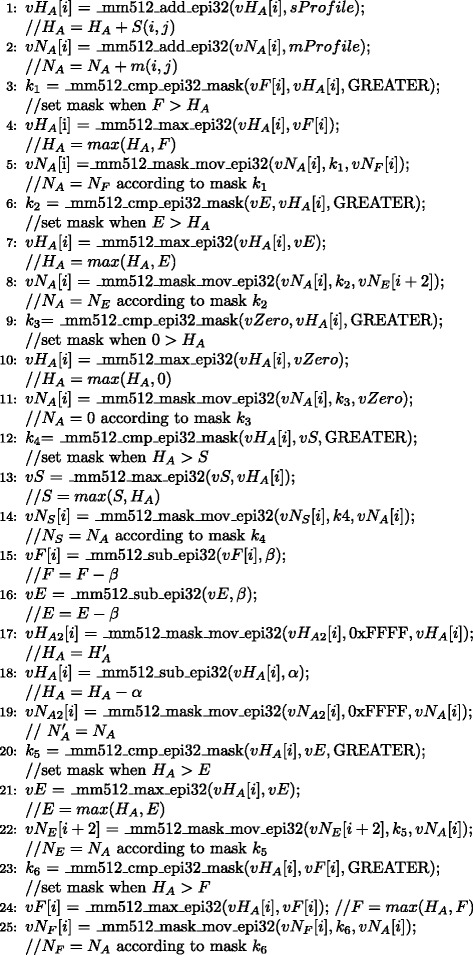
Xeon Phi vectorized implementation of pairwise alignment according to Definition 2 by dynamic programming using 25 core instructions. The variables in these instructions can be divided into two classes. One class includes *v* *H*_*A*_, *vE*, *vF*, and *vS* which are used in the Smith-Waterman algorithm. Another class contains *v* *N*_*A*_, *v* *N*_*E*_, *v* *N*_*F*_ and *v* *N*_*S*_ which are defined in Definition 2. Here *v* *N*_*A*_ is the target vector and *v* *N*_*S*_ is the value *n* *i* *d*(*S*_*i*_,*S*_*j*_)

Input data set sizes for MSA are typically smaller than for database search (protein sequence databases typically contain may millions of sequences while large-scale MSAs are computed for a few thousand protein sequences) making the subject sequence set for distance matrix computation comparatively small. In order to design an efficient parallel distance matrix computation algorithm on Xeon Phi, we have used the task partitioning method shown in Fig. 3. In our method, the sequences are sorted by their lengths and then partitioned into smaller sized batches. In an alignment task, a query sequence will be aligned to the corresponding sequence batch. This procedure will continue until all task batches are calculated. We have implemented the whole process into two parallel parts: the thread level and the VPU level. On the thread level, the process aligning *S*_*i*_ to *S*={*S*_(*i*+1)_,…,*S*_*n*_} is grouped to *task*_*i*_, and each task is processed by a thread. On the VPU level, multi-pairwise comparisons are performed in parallel on VPUs. In our method, *S*={*S*_(*i*+1)_,…,*S*_*n*_} is packed into a 2D buffer which has 16 channels, meaning that sequence *S*_*i*_ can be aligned to 16 different sequence in the 16-channel buffer in parallel. We have used Knights Corner instructions to implement this part. Figure 4 shows the pseudo-code of our algorithm framework. In order to take advantage of both CPUs and Xeon Phis in a node to process MSA for large-scale datasets, we have implemented our algorithm framework using the offload model. We have implemented the arithmetic operations specified by the equations in Definition 2 using a number of Knights Corner instructions (see Fig. 5) for Xeon Phis. These instructions are executed on VPUs to calculate the sixteen residue vectors of alignment matrices according to Definition 2. For CPUs, VPUs fetch 8 residues each time. The core instructions used on CPUs are identical with Xeon Phis, whereas they have been implemented using different 256-bit AVX intrinsic instructions.

**Fig. 6:**
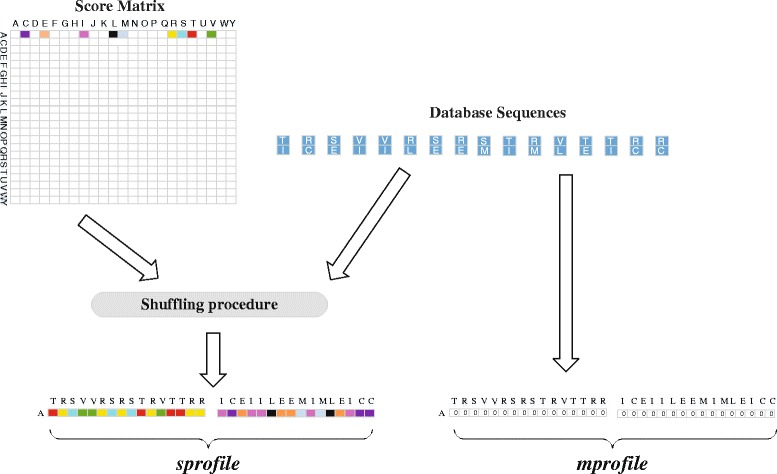
An example of how to create the *sprofile* and the *mprofile* for two sequence vectors to match the ‘A’ residue

Before performing the alignment process, two temporary score vectors (the *sprofile* and the *mprofile* in Fig. 4) are created to help improve the IO efficiency for loading the substitution matrix values and the *m*(*i,j*) (see Definition 2) values in parallel. Figure 6 shows an example of how to create these two temporary vectors. From Fig. 6 we can see that the substitution score matrix, the current database sequence vector, and the query sequence will be used to create the *sprofile* and the *mprofile*. VPUs will make use of these two score vectors to load substitution values and *m*(*i,j*) values quickly. The shuffling procedure in Fig. 6 is used to help VPUs fetch corresponding values from the substitution matrix in parallel [7].

In our implementation, the size of these two temporary vectors for Xeon Phi and CPU is 16 and 8 separately.

**Fig. 7:**
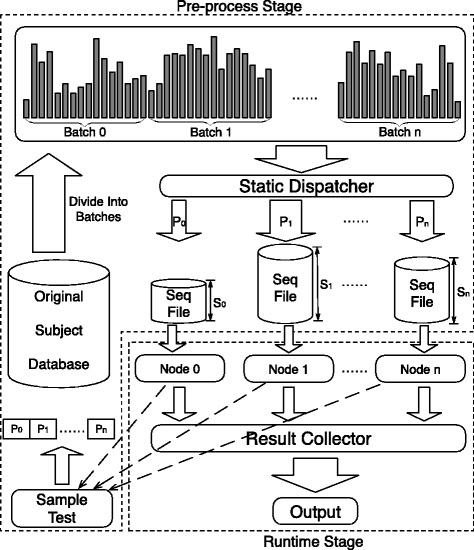
Our device level dynamic task distribution framework. The *black dots* denote tasks

We have designed and implemented a device level dynamic task distribution framework to distribute tasks to both the CPU device and the Xeon Phi device. Figure 7 shows our framework. In this framework, the task distributor is implemented as a critical section to prevent the concurrent access to shared tasks. It is also used to perform the dynamic distribution of tasks to CPUs and Xeon Phis. In Fig. 7, both CPUs and Xeon Phis fetch and process multiple tasks in parallel. After the allocated tasks are processed, both devices will send requirements to the data distributor to request for new tasks. All new task requirements will first be identified and queued by the data distributor. It then distributes tasks to the queue in order.

## Cluster level data parallelization

Our approach is based on the fact that both subject database batches (for database searching) and MSA tasks can be scanned in parallel. Thus we have implemented the cluster level data parallel algorithm for these two alignment applications. The cluster level data parallel algorithm is encoded on the master node. The master-node partitions the subject database or the MSA tasks into a number of chunks that will be sent to different compute nodes. Our approach is implemented using the following modules:

- **Dispatcher (Master):** Partitions subject database or MSA tasks into a number of chunks in a preprocessing steps and sends them to compute nodes.
- **Algorithms on a Single Node (Worker):** Receives sequence chunks from master and performs the corresponding DP calculations.
- **Result Collector (Master):** Performs additional operations required to further process the returned results.

### Protein sequence database search

**Fig. 8:**
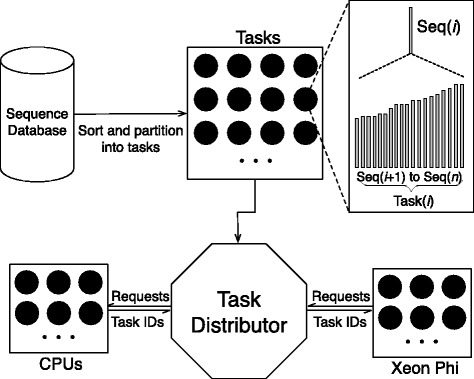
Illustration of our method to dispatch database subsets to all nodes. The node who has more computing power will be dispatched more sequences, which will finally balance the workload at runtime

In our work, we have implemented a static dispatcher for our cluster level parallel database searching algorithm. Figure 8 illustrates our method. In Fig. 8, the static dispatcher in the preprocess stage first divides the database into several chunks with respect to the total number of nodes. The database chunks are then sent to the corresponding node for local searching. Since the compute power of all compute nodes may vary, the size of each database subset can also vary. In order to achieve load balancing among all nodes, we have implemented a sample test method. In our method, at the preprocess stage (see Fig. 8), firstly a sample test is performed to explore the compute power of all compute nodes. Performance factors of different nodes are then automatically generated. In our work, we name this factor the compute power *P*_*i*_ for node *i*. With the performance factor *P*_*i*_, we can then calculate the appropriate size of the database subset allocated to node *i*.

### MSA

**Fig. 9:**
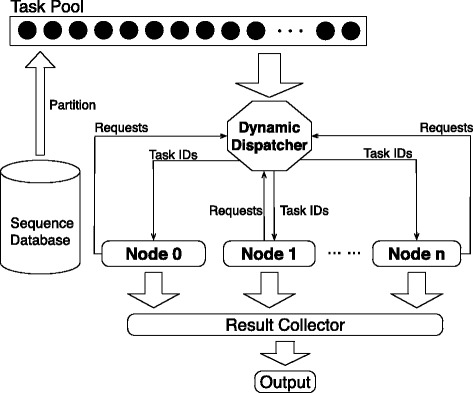
Illustration of our method to dispatch tasks dynamically to all nodes. The task partition method is illustrated in Fig. 3

We have designed and implemented a cluster level dynamic dispatcher to distribute tasks to compute nodes. Figure 9 illustrates our method. In this method, the dynamic dispatcher first divides the dataset into a set of tasks which are organized as a task pool. Then, multiple tasks are sent to each node for local distance matrix computation. After the allocated tasks are processed, each node will send requirements to the dispatcher to ask for new tasks to process. This procedure will continue until all tasks are processed.

## Results and discussion

### Test platforms

We have implemented the proposed methods using C++ and evaluated them on compute nodes with the following Xeon Phi cards (with ECC enabled) installed:

- *Intel Xeon Phi 7110P*: 61 hardware cores, 1.1 GHz processor clock speed, 8 GB GDDR5 device memory.
- *Intel Xeon Phi 31S1P*: 57 hardware cores, 1.1 GHz processor clock speed, 8 GB GDDR5 device memory.

Tests have been conducted on a Xeon Phi cluster with three compute nodes that are connected by an Ethernet switch. There are two Xeon E5 CPUs and 16GB RAM on each compute node. The cluster runs Centos 6.5 with the Linux kernel 2.6.32-431.17.1.el6.x86_64. The CPU configuration on each node varies, as is listed in Table 1. We also have SSD hard disks installed on each compute node.

**Table 1:** Test cluster configurations

Node	CPU	Coprocessor
*N*_1_	Xeon E5-2620 (6 cores) * 2	Xeon Phi 7110p * 1
*N*_2_	Xeon E5-2620v2 (6 cores) * 2	Xeon Phi 7110p * 2
*N*_3_	Xeon E5-2650v2 (8 cores) * 2	Xeon Phi 31s1p * 4

### Protein sequence database search

A performance measure commonly used in computational biology to evaluate Smith-Waterman implementations is *cell updates per second* (*CUPS*). A CUPS represents the time for a complete computation of one entry of the DP matrix, including all comparisons, additions and maxima operations.

We have scanned three protein sequence databases: (i) the 7.5 GB UniProtKB/Reviewed and Annotated (5,943,361,275 residues in 16,110,751 sequences), (ii) the 18 GB UniProtKB/TrEMBL (13,630,914,768 residues in 42,821,879 sequences), and (iii) the 37 GB merged Non-Redundant plus UniProtKB/TrEMBL (24,323,686,690 residues in 73,401,766 sequences) for query sequences with varying lengths. Query sequences used in our tests have the accession numbers P01008, P42357, P56418, P07756, P19096, P0C6B8, P08519, and Q9UKN1.

#### Performance on a single node

We have firstly compared the single-node performance of our methods to SWAPHI [8] and CUDASW++ 3.1 [26]. SWAPHI is another parallel Smith-Waterman algorithm on Xeon Phi-based neo-heterogeneous architectures. It is also implemented using the offload model. However, SWAPHI can only run search tasks on Xeon Phi; i.e. it does not exploit the computing power of multi-core CPUs. SWAPHI cannot handle search tasks for large-scale biological databases. In our tests, we find that the database size limitation for SWAPHI is less than the available RAM size; i.e. 16 GB. CUDASW++ 3.1 is currently the fastest available Smith-Waterman implementation for database searching. It makes use of the compute power of both the CPU and GPU. At the CPU side, CUDASW++ 3.1 carries out parallel database searching by invoking the SWIPE [18] program. It employs CUDA PTX SIMD video instructions to gain the data parallelism at the GPU side. The database size supported by CUDASW++ 3.1 is less than the memory size available on the GPU. Neither SWAPHI nor CUDASW++ 3.1 supports clusters.

For single-node tests, we have used the *N*_2_ node (see Table 1) as test platform. In our experiments, we run our methods with 24 threads on two Intel E5-2620 v2 six-core 2.0 GHz CPUs and 240 threads on each Intel Xeon Phi 7110P respectively. We execute SWAPHI with 240 threads on each Xeon Phi 7110P. We have executed CUDASW++ 3.1 on another server with the same two Intel E5-2620 v2 six-core 2.0 GHz CPUs plus two Nvidia Tesla Kepler K40 GPUs with ECC enabled. 24 CPU threads are also used for CUDASW++ 3.1. If not specified, default parameters are used for both SWAPHI and CUDASW++ 3.1. Furthermore, all available compiler optimizations have been enabled. The parameters *α*=10, and *β*=2 have been used in our experiments. The substitution matrix used is BLOSUM62.

**Fig. 10:**
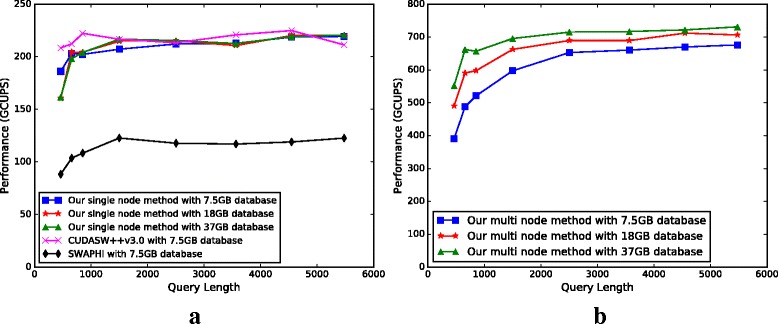
**a** performance comparison on a single node (*N*_2_) between our method, CUDASW++v3.1 and SWAPHI. **b** performance results of our method using all three compute nodes

We have measured the time to compute the similarity matrices to calculate the *computing CUPS* values in our experiments. Figure 10a shows the corresponding computing GCUPS values of our methods, SWAPHI and CUDASW++ 3.1 for searching the 7.5 GB UniProtKB/Reviewed and Annotated protein database using different query sequences. From Fig. 10a we can see that the computing GCUPS of our multi-pass method is comparable to CUDASW++ 3.1. Both of them achieve better performance than SWAPHI.

SWAPHI and CUDASW++ 3.1 cannot support search tasks for the 18 GB and 37 GB databases. Thus, we only use our methods to search them. Figure 10a also reports the performance of our methods for searching these two databases. The results show that our methods can handle large-scale database search tasks efficiently.

#### Performance on a cluster

Figure 10b shows the performance of our methods using all three cluster nodes. The result indicates that our methods exhibit good scalability in terms of sequence length and size, and number of compute nodes. Our method achieves a peak overall performance of 730 GCUPS on the Xeon Phi-based cluster.

### MSA

A set of performance tests have been conducted using different protein sequence datasets to evaluate the processing time for the distance matrix computation step of our implementation in comparison to MSA-CUDA [32]. The datasets are extracted from the UniProtKB/Reviewed database, whose details are listed in Table 2. We have used two groups of datasets in our tests. Datasets *S*_1_ to *S*_6_ are used to compare the performance of our method and MSA-CUDA, where the sequence numbers are small since MSA-CUDA can not handle datasets with large sequence number. Datasets *L*_1_ to *L*_6_ are used to evaluate the performance of our method for handling large-scale datasets. These datasets consist at least 10,000 sequences.

**Table 2:** Test datasets for MSA

Dataset	Avg. Length	#Sequences	Workload (GCells)
*S*_1_	465	200	4.35
*S*_2_	472	400	17.84
*S*_3_	474	600	40.52
*S*_4_	476	800	72.56
*S*_5_	476	1000	113.54
*S*_6_	480	1200	164.13
*L*_1_	150	30000	10891
*L*_2_	382	16000	18692
*L*_3_	935	10000	39148
*L*_4_	274	40000	60246
*L*_5_	1350	10000	88013
*L*_6_	700	24000	133112

The workload for computing a distant matrix grows quadratically with respect to the number of input sequences. The average sequence length of the dataset also has a great impact on the computing workload. We have used the following equation to measure the workload needed to process a dataset.

$$ W = \sum\limits_{i=1}^{n} \left(L_{i} * \sum\limits_{j=i+1}^{n} L_{j}\right) $$

where *L*_*i*_ denotes the length of the *i*th sequence in the dataset. Thus, the workload *W* is actually the total number of matrix cells to be calculated. As our method utilizes the constant 25 instructions for calculating each cell (as is listed in Fig. 5), the execution time grows linearly with *W*. Table 2 also lists the workload needed for processing each dataset.

#### Performance for processing medium-scale datasets

**Fig. 11:**
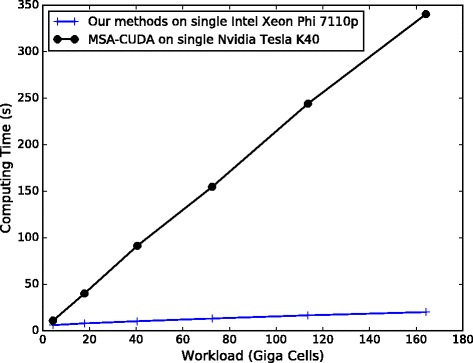
Runtime (in seconds) for processing datasets *S*_1_ to *S*_6_. Our method runs on a Xeon Phi 7110P. MSA-CUDA runs on a Tesla K40 GPU

For the medium-scale datasets *S*_1_ to *S*_6_, MSA-CUDA is benchmarked on a Tesla K40 GPU with default options and all available compiler optimizations enabled. Our implementation runs on an Intel Xeon Phi 7110P with 240 threads. Figure 11 shows the performance comparison between our method and MSA-CUDA. From Fig. 11 we can find our implementation achieves significantly better performance compared to MSA-CUDA.

#### Performance for processing large-scale datasets

**Fig. 12:**
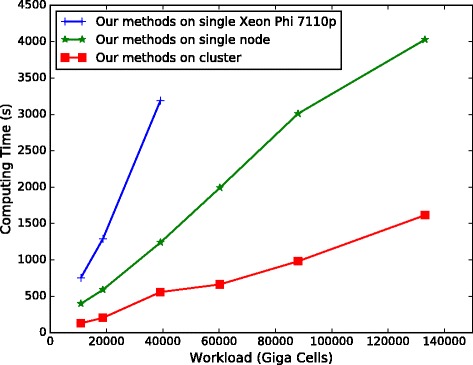
Runtime (in seconds) for processing datasets *L*_1_ to *L*_6_. We have run our method on an Intel Xeon Phi 7110P, the *N*_2_ node and the cluster, respectively

For the large-scale datasets *L*_1_ to *L*_6_, MSA-CUDA cannot work normally. We have run our methods on a single Intel Xeon Phi 7110P, the *N*_2_ node and the cluster respectively. The performance results are shown in Fig. 12. Figure 12 indicates that our methods exhibit very good scalability in terms of workload and number of compute nodes. Although the nodes in our cluster have different compute power, our dynamic task dispatching scheme still works efficiently. Moreover, our method on the cluster is able to process large-scale datasets that are rarely seen in other MSA implementations, whereas the runtime is still acceptable.

## Conclusion

We have presented two parallel algorithms for protein sequence alignment based on the dynamic programming concept which can be efficiently mapped onto Xeon Phi clusters. Our methods exhibit good performance on a single compute node as well as good scalability in terms of sequence length and size, and number of compute nodes for both protein sequence database search and distance matrix computation employed in multiple sequence alignment. Furthermore, the achieved performance is highly competitive in comparison to other optimized Xeon Phi and GPU implementations. Biological sequence databases are continuously growing establishing the need for even faster parallel solutions in the future. Hence, our results are especially encouraging since performance of many-core architectures grows much faster than Moore’s law as it applies to CPUs. For instance, the performance improvement with at least a factor of 3 can be expected on the already announced next-generation Xeon Phi product.
